# Microdissection of lampbrush chromosomes as an approach for generation of locus-specific FISH-probes and samples for high-throughput sequencing

**DOI:** 10.1186/s12864-016-2437-4

**Published:** 2016-02-20

**Authors:** Anna Zlotina, Tatiana Kulikova, Nadezda Kosyakova, Thomas Liehr, Alla Krasikova

**Affiliations:** Department of Cytology and Histology, Saint Petersburg State University, Saint Petersburg, Russia; Institute of Human Genetics, Jena University Hospital, Friedrich Schiller University, Jena, Germany

**Keywords:** Chromosome microdissection, Chicken, Lampbrush chromosomes, Special loops, Locus-specific FISH-probes, Genomic mapping, Next-generation sequencing

## Abstract

**Background:**

Over the past two decades, chromosome microdissection has been widely used in diagnostics and research enabling analysis of chromosomes and their regions through probe generation and establishing of chromosome- and chromosome region-specific DNA libraries. However, relatively small physical size of mitotic chromosomes limited the use of the conventional chromosome microdissection for investigation of tiny chromosomal regions.

**Results:**

In the present study, we developed a workflow for mechanical microdissection of giant transcriptionally active lampbrush chromosomes followed by the preparation of whole-chromosome and locus-specific fluorescent in situ hybridization (FISH)-probes and high-throughput sequencing. In particular, chicken (*Gallus g. domesticus*) lampbrush chromosome regions as small as single chromomeres, individual lateral loops and marker structures were successfully microdissected. The dissected fragments were mapped with high resolution to target regions of the corresponding lampbrush chromosomes. For investigation of RNA-content of lampbrush chromosome structures, samples retrieved by microdissection were subjected to reverse transcription. Using high-throughput sequencing, the isolated regions were successfully assigned to chicken genome coordinates. As a result, we defined precisely the loci for marker structures formation on chicken lampbrush chromosomes 2 and 3. Additionally, our data suggest that large DAPI-positive chromomeres of chicken lampbrush chromosome arms are characterized by low gene density and high repeat content.

**Conclusions:**

The developed technical approach allows to obtain DNA and RNA samples from particular lampbrush chromosome loci, to define precisely the genomic position, extent and sequence content of the dissected regions. The data obtained demonstrate that lampbrush chromosome microdissection provides a unique opportunity to correlate a particular transcriptional domain or a cytological structure with a known DNA sequence. This approach offers great prospects for detailed exploration of functionally significant chromosomal regions.

**Electronic supplementary material:**

The online version of this article (doi:10.1186/s12864-016-2437-4) contains supplementary material, which is available to authorized users.

## Background

Along with conventional cytogenetic and cytological techniques, chromosome microdissection represents a powerful approach for the investigation of chromosomes and their regions. On the contrary to other chromosome isolation methods like flow-sorting or laser capture chromosome microdissection, mechanical (glass needle-based) chromosome microdissection allows to dissect chromosome fragments as small as a single chromosome band [1]. Dissected chromosomal material can be successfully amplified and used as a molecular probe for in situ hybridization. Thus, glass needle-based microdissection helps to obtain broad panels of locus-specific and whole-chromosome painting probes marking different chromosomal regions of various length and organization complexity (including the regions enriched by highly-repetitive DNA). Such probes are widely used to identify the chromosomes, to map precisely the evolutionary and clinical chromosomal breakpoints, as well as to determine the nature of marker and derivative chromosomes [2–10]. In the field of comparative cytogenetics the probes derived by chromosome microdissection are indispensable for revealing the orthologous regions in karyotypes of species belonging to different taxonomic groups [11–19]. Apart from this, the probes prepared by microdissection allow to localize individual chromosomes and their specific regions in interphase nuclei [20–22]. Recently it became possible to analyze dissected chromosomal regions using next-generation sequencing (NGS) technologies [23, 24] which offer great prospects for more detailed research of specific loci.

However, despite the advantages, the conventional chromosome microdissection still has some limitations. In vast majority of cases, the biomaterial subjected to microdissection procedure is highly compacted mitotic metaphase chromosomes with an average size of several microns for vertebrates. Dissection of microchromosomes or regions of interest smaller then a single G-band proved to be tricky [25]. In addition, microdissection procedure requires the unambiguous identification of a target chromosome region [26]. Finally, though it was shown that chromosome paints can be obtained based on a single dissected chromosome, it is recommended to collect no less than 10–20 dissected copies of the target region for further successful amplification and establishing of a representative DNA library [25].

Due to these limitations, standard microdissection of mitotic chromosomes does not seem to be a method of choice for the appropriate analysis of tiny chromosomal regions. However, use of extended lampbrush chromosomes (LBCs) as an initial material for microdissection could help to overcome the abovementioned limitations. Lampbrush chromosomes are meiotic half-bivalents from growing oocytes of some animals, including birds, represented by an array of distinct chromomeres with laterally extended loops [27–29]. Such chromosomes are highly decondensed because of intensive transcriptional activity. In average, avian lampbrush chromosomes are 20–30 times longer than corresponding mitotic metaphase chromosomes that makes the microdissecion procedure much simpler. Lampbrush chromosomes bear unusual marker structures including so-called «complex loops», whose organization, precise genomic position, molecular composition and functions are still obscure [27–32]. The utilization of microdissected material as probes for in situ hybridization, as well as its further analysis with deep-sequencing approaches, could be a promising tool for all-round cytogenetic and cytological exploration of such marker structures.

At present there are only single studies where microdissection of lampbrush chromosomes was applied. In particular, regions bearing so-called protein bodies associated with chaffinch lampbrush chromosomes were dissected, the isolated material being amplified and used for Southern-blotting [33]. Penrad-Mobayed and co-authors dissected globular and granular complex marker loops from lampbrush chromosomes of the newt *Pleurodeles waltl* [34]. These loops were shown to produce transcripts of moderately repeated DNA elements, which, however, proved to be non-locus-specific and were detected in multiple chromosomal regions. Thus, up to date there is no approach to microdissection of whole lampbrush chromosomes or small lampbrush chromosome regions for generation of highly specific FISH-probes and for further analysis of the isolated material by NGS technologies.

In present work, we developed an approach for lampbrush chromosome microdissection, DNA or cDNA amplification from the isolated material followed by highly specific FISH-probes generation and high-throughput sequencing. In particular, the chicken (*Gallus g. domesticus,* GGA) chromosomal regions as small as a single chromomere and individual pairs of simple loops were successfully dissected, amplified and used for FISH and NGS, with only one bivalent copy being taken as input material. Such a comprehensive approach allows to assign unambiguously the position of individual chromomeres and cytological markers of lampbrush chromosomes to genomic coordinates.

## Methods

### Chromosome preparation

Chicken lampbrush chromosomes (LBCs) were manually isolated from growing oocytes with a diameter of 0.5-1.5 mm as described elsewhere [35]. All institutional and national guidelines for the care and use of laboratory and farm animals were followed. The animal studies received approval of the Ethical committee of Saint-Petersburg State University. Preparations were fixed in 2 % formaldehyde for 30 min, dehydrated in ethanol and air-dried. For microdissection procedure only freshly prepared slides with chromosomes were used (within 2–4 days after fixation). To avoid any contamination events, the instruments and the solutions for chromosome isolation were autoclaved; all manipulations were carried out in sterile laboratory gloves. Mitotic metaphase chromosomes were obtained from chicken embryonic fibroblasts according to conventional protocols.

### Needle-based microdissection and degenerate oligonucleotide-primed PCR

Glass needle-microdissection was performed according to the previously published protocol [25] with some modifications. Due to the size of lampbrush chromosomes, objective lenses with the magnification of × 10 and × 20 were used to visualize target chromosomes. Lampbrush chromosomes preparations were not stained, and microdissection targets were identified based on the phase contrast images. In some cases tips of microdissection needles of a standard size were broken to slightly increase their diameter. Microdissected fragments were transferred into micropipettes containing collection drop solution (30 % glycerol, 10 mM Tris/HCl, pH 7.5, 10 mM NaCl, 0.1 % SDS, 1 mM EDTA, 0.1 % Triton X-100, 1.44 mg/ml proteinase K) and incubated in a humidified tray at 60 °C for 1–2 h.

After that the dissected chromosomal material was transferred into microtubes containing 0.60 μl Sequenase buffer (USB), 0.40 μl of 0.2 mM dNTPs, 0.63 μl of 40 mM DOP primer (degenerate oligonucleotide primer, 5′-CCG ACT CGA GNN NNN NAT GTG G-3′) and 3.37 μl of PCR water per sample. DOP-PCR (degenerate oligonucleotide-primed PCR, [36]) was performed as previously described [25] with minor modifications. Eight low-annealing temperature amplification cycles with Sequenase Version 2.0 DNA Polymerase (Affymetrix/USB) were followed by adding 45 μl of PCR mix for further 30 high annealing temperature cycles (27.03 μl PCR water, 10.00 μl 5xPCR buffer, 4.40 μl 2,5 M dNTPs mix, 2.5 μl 50 mM MgCl2, 0.20 μl Platinum Tfi Exo(−) polymerase (Invitrogen)).

Three microliters of the primary DOP-PCR product of the samples and of a collection drop without DNA material as a negative control were run in 2 % agarose gel to test the efficiency of the amplification.

### Reverse transcription

RNA-containing marker structures were dissected from 6 correspondent lampbrush bivalents and collected into one Pasteur pipette with a collection drop, containing 1.44 mg/μl of proteinase K. Content of the collection drop was slightly modified as compared to DNA microdissection: SDS and Triton-X100 were excluded and 5U/μl of RiboLock (Thermo Scientific) were added. The Pasteur pipette with a collection drop containing the dissected material and a pipette with a collection drop only (negative control) were incubated at 60 °C for 1–2 h in humidified tray. Then collection drops were transferred into two tubes with 8 μl of nuclease-free water (Thermo Scientific). To inhibit proteinase K activity the tubes were heated to 94 °C for 5 min and then chilled on ice. DNAse I solution (DNAse I buffer, 0.1 U/μl DNAse I, 1 U/μl DNAse I) was added to each tube for total volume 10 μl, after that the tubes were incubated at 37 °C for 30 min. DNAse I in the reaction mix was inactivated by heating at 65 °C for 10 min followed by chilling on ice. To prevent RNA degradation during DNAse I inactivation, 2.6 mM of EDTA were added before heating. Content of each tube was aliquoted into two for a negative control without reverse transcriptase. Then the components of reverse transcription reaction (nuclease-free water, reaction buffer, 10 ng/μl random prime hexamer, 1 mM dNTP, reverse transcriptase 10 U/μl, RiboLock 1 U/μl) were added for total volume 20 μl. Reverse transcription reaction was performed according to the manufacturer’s recommendations (ThermoScientific). Specifically, the tubes were pre-incubated for 5 min at 25 °C and then incubated at 42 °C for 60 min. Reaction was terminated by heating at 70 °C for 5 min. Reverse transcription products were amplified by DOP-PCR under the same conditions as DNA dissected material. In this case, 3.37 μl of reverse transcription product were used as a template in PCR mix in the low annealing temperature amplification cycles.

### Fluorescent in situ hybridization

#### Probes preparation

The primary DOP-PCR products were labeled with haptens – biotin or digoxigenin – by PCR with the same degenerate universal primer [36]. The reaction mix contained 1 × Taq-polymerase buffer (Sileks), 2.5 mM MgCl_2_, 0.2 mM dNTP, 0.5 pM primer, 1–2 U of Taq-polymerase (Sileks). Two-five microliters of the primary PCR products were added as a template, with total volume being 20 μl. PCR was performed at standard conditions with 30 cycles and with primer annealing temperature 55 °C.

To evaluate the yield of the amplification, 0.5-1.0 μl of labeled PCR products were run in 1 % agarose gel. The efficiency of labeling was evaluated by spotting the probe dilutions ranging from 100 pg/μl to 0.1 pg/μl onto a nylon membrane strip followed by a detection procedure with streptavidin or anti-digoxigenin antibodies conjugated with alkaline phosphatase [37].

Labeled PCR products were dissolved in a standard hybridization buffer (50 % deionized formamide (ICN), 2× saline-sodium citrate (1× SSC: 0.15 M NaCl, 15 mM Na_3_C_6_H_5_O_7_), 10 % dextran sulphate (Pharmacia Biotech)) to a final concentration of 20–40 ng/μl with a 50-fold excess of salmon sperm DNA (Invitrogen).

#### In situ *hybridization*

Fluorescent in situ hybridization was applied to mitotic metaphase and lampbrush chromosomes. Metaphase chromosomes were pre-treated with pepsin (0.01 % in 0.01 N HCl) and post-fixed with 1 % formaldehyde in PBS according to the standard procedure. FISH on lampbrush chromosomes was carried out according to a DNA/(DNA + RNA) hybridization protocol without any chromosome pretreatments. Chromosomes and probes were jointly denatured on a slide covered with a coverslip at 82 °C for 5 min followed by hybridization at 37 °C in a humid chamber for 16–20 h. Then, the slides were washed in two changes of 0.2 × SSC at 60 °C and two changes of 2 × SSC at 45 °C. Avidin-Alexa 488 (Molecular Probes Inc.), Avidin-Cy3 (Jackson Immuno Research Laboratories) and mouse antibody against digoxigenin conjugated with Cy3 (Jackson ImmunoResearch Laboratories) were used to detect biotin- and digoxigenin-labeled probes, respectively. For signal amplification, we carried out additional incubation with biotinylated anti-avidin (Vectorlabs) followed by the second round of incubation with avidin-Cy3 or avidin-Alexa 488 for biotin-labeled probes, and incubation with Cy3-conjugated goat anti-mouse IgG + IgM (H + L) (Jackson ImmunoResearch Laboratories) for digoxigenin-labeled probes. All preparations were dehydrated, air-dried, and mounted in antifade solution containing 1 μg/ml 4,6-diamidino-2-phenylindole (DAPI).

### Library preparation and high throughput sequencing

The 454 library preparation was performed according to the manufacturer’s protocol with some modifications. For additional reamplification and introduction of the sequencing 454-adapters to primarily amplified microdissected material, we used a standard PCR with 35 cycles with the following three pairs of primers:

- A-LibA1 (forward) CGTATCGCCTCCCTCGCGCCA-TCAG-ACACGACGACT-CCGACTCGAG
- B-LibA1 (reverse) CTATGCGCCTTGCCAGCCCGC-TCAG-ACACGACGACT-CCGACTCGAG
- A-LibA2 (forward) CGTATCGCCTCCCTCGCGCCA-TCAG-ACACGTAGTAT-CCGACTCGAG
- B-LibA2 (reverse) CTATGCGCCTTGCCAGCCCGC-TCAG-ACACGTAGTAT-CCGACTCGAG
- A-LibA3 (forward) CGTATCGCCTCCCTCGCGCCA-TCAG-ACACTACTCGT-CCGACTCGAG
- B-LibA3 (reverse) CTATGCGCCTTGCCAGCCCGC-TCAG-ACACTACTCGT-CCGACTCGAG

Each sample was barcoded with an individual adapter per run. The reaction mix contained Taq-polymerase buffer (Sileks), 2.5 mM MgCl_2_, 0.2 mM dNTP, 0.2 pM LibA-A and LibA-B primers, 2 U of Taq-polymerase (Sileks), 2–3 μl of primarily amplified microdissected material with total volume 50 μl. The PCR-products were run in a 2 % agarose gel from which the fragments of size above 300 bp were eluted to get rid of short length fragments and primer dimers. The concentration of samples after the elution was 4–10 ng/μl.

Sequencing runs were carried out with 454/Roche GS Junior genome sequencer according to the manufacturer’s instructions; single-end sequencing was performed. Each experiment was processed in a 1/3 run.

### Data analysis

The NGS data was processed and analyzed using the web-based bioinformatic platform Galaxy [38–40]. In particular, input files were converted to an appropriate FASTQ format using sff converter (version 1.0.1) and FASTQ Groomer (version 1.0.4) tools; after that the quality of sequence data was evaluated using FastQC tool. All reads were trimmed to get rid of terminal primer/adapter sequences and to remove poor quality base calls from the end (FASTX – toolkit). Additionally, the sequences were also filtered by length with final read length being about 200 bp. To map the sequences to chicken reference genome assembly (chicken Nov. 2011 ICGSC Gallus_gallus-4.0/galGal4), a short-read aligner Bowtie2 was applied [41]. To evaluate the quality and basic statistic characteristics of the alignment data (such as total number of reads, number of mapped and unmapped reads, coverage, GC content, chromosome distribution) BAM files were analyzed with Qualimap v.2.1 tool [42]. The data visualization and analysis were carried out with genome browser Integrative Genomics Viewer (IGV) [43, 44]. The mapped chromosome regions were also evaluated with regard to some genome characteristics (such as gene-density, repeats content) using corresponding imported tracks downloaded from the UCSC Genome Browser (http://genome.ucsc.edu).

## Results and discussion

### Microdissection of individual chromomeres and lateral loops from chicken lampbrush chromosomes

**Fig. 1:**
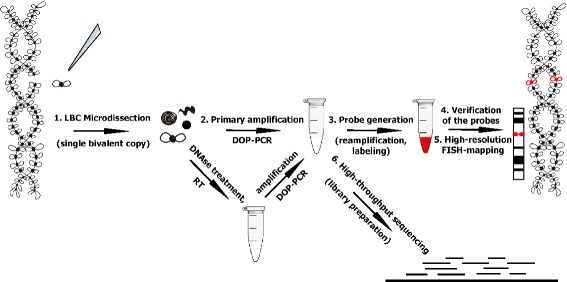
The scheme illustrating the developed workflow of lampbrush chromosomes (LBC) microdissection. The main steps are as follows: 1. Mechanical microdissection of lampbrush chromosome regions; 2. Primary amplification of isolated DNA material by DOP-PCR. Alternatively, for investigation of RNA-component of chromosomal loci, before the amplification the dissected material was pretreated with DNAse I followed by reverse transcription; 3. FISH-probe generation via reamplification and labeling; 4. Verification of specificity and brightness of the probes by FISH on metaphase chromosomes; 5. High-resolution FISH-mapping of dissected regions on lampbrush chromosomes; 6. Preparation of DNA-libraries for high-throughput sequencing. Processing, visualization and analysis of the sequencing data using web-based bioinformatic platform Galaxy [39] and genome browser IGV [43, 44]. The sequence of actions is depicted by arrows

**Fig. 2:**
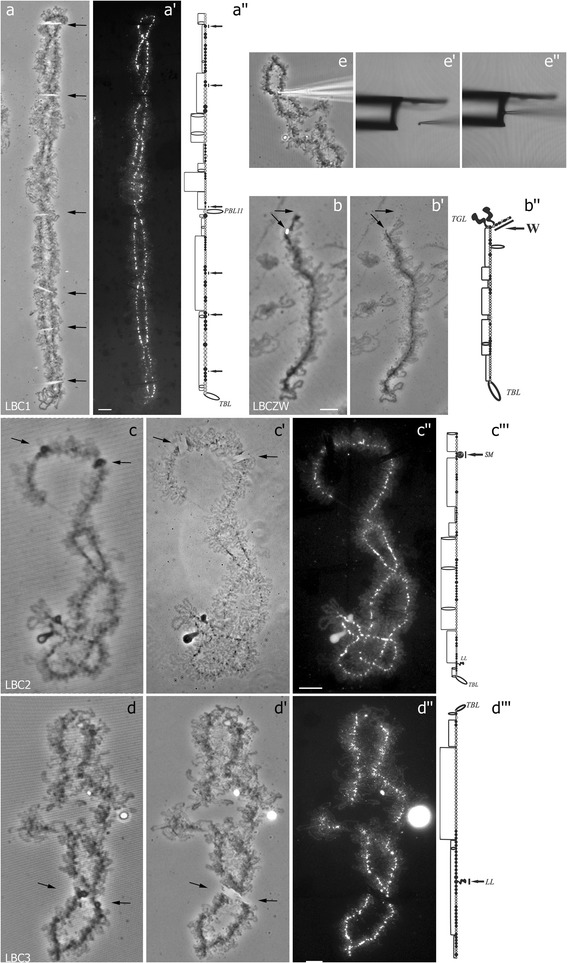
Microdissection of different regions from chicken lampbrush chromosomes (examples). Microdissection of single chromomeres from LBC1 (**a**, *a’*), whole chromosome W from the sex bivalent (**b**, *b*’), the structure «Spaghetti marker» (SM) from LBC2 (**c**-*c”*) and the special loop «Lumpy loop» (LL) from LBC3 (**d**-*d”*). (**b**, **c**, **d**) – phase contrast images of corresponding lampbrush chromosomes before microdissection procedure. (**a**, *b’*, *c’*, *d’*) – phase contrast images of corresponding lampbrush chromosomes after microdissection procedure. Black arrows point to the regions subjected to microdissection. (*a’, c”, d”*) – images of corresponding lampbrush chromosomes are counterstained with DAPI. (*a”, b”, c”’, d”’*) – Cytological maps of chromomere-loop patterns [53] showing dissected regions for LBC1, ZW bivalent, LBC2, LBC3, respectively. (**e**-*e”*) Transfer of microdissected material with a glass-needle into a Pasteur pipette containing a collection drop. Scale bar = 10 μ. PBL11, TGL, TBL-marker loops

Here we developed a workflow for lampbrush chromosome microdissection followed by locus-specific FISH-probe generation and high-throughput sequencing (Fig. 1). For microdissection procedure, we chose the largest chicken lampbrush macrochromosomes 1, 2 and 3 (LBC1, 2, 3, respectively). Individual chromomeres of various size were dissected from lampbrush chromosome 1, and the material of marker structures was isolated from lampbrush chromosomes 2 and 3 (Fig. 2a-a’, c-c”, d-d”). Positions of all dissected regions were assigned to cytological maps of chromomere-loop pattern of corresponding lampbrush chromosomes (Fig. 2a”, c’”, d’”). As a control experiment, microdissection of whole lampbrush chromosome W (LBCW) from the sex bivalent ZW was carried out (Fig. 2b-b”). The dissection procedure is demonstrated in Fig. 2e-e”.

Six different chromomeres were dissected along a single lampbrush chromosome 1 (Fig. 2a-a”), each of which was then transferred into an individual Pasteur pipette with a collection drop. Thus, only one bivalent copy was used as a starting material for lampbrush chromosome microdissection. Both prominent loopless chromomeres and small chromomeres with extended pairs of lateral loops were taken into analysis. Here we expected to isolate chromosome regions as small as a couple of megabases in size, given that the average DNA content per single chromomere in chicken lampbrush macrobivalents has been estimated to be 1.5-2 Mb [45]. Such chromosomal regions are about five times smaller than an average G-band of metaphase chromosomes often used for standard microdissection procedure.

On the example of two types of cytological marker structures that appear on chicken lampbrush chromosomes 2 and 3, we developed an approach to the identification of the loci of their formation. The material of the so-called «spaghetti marker» (SM) and «lumpy loops» (LLs) was dissected from the short arm of lampbrush chromosome 2 and the long arm of lampbrush chromosome 3, respectively (Fig. 2c-c”’, d-d”’). Spaghetti marker represents a morphologically prominent structure of 2–5 μm in size that is largely composed of protein fibers [31], while lumpy loops represent compact globular «special loops» with a complex morphology [30]. As in case of chromomeres, individual marker structures were dissected from six LBC2 and six LBC3 into separate microtubes. For comparison, in a previously published study, for isolation of DNA from «globular» and «granular» landmark loops (bivalent VII) of *P. waltl*, as much as 500 loop pairs were scratched and used as a starting material for subsequent microcloning [34].

The next crucial step of the procedure was the primary amplification of the dissected material by PCR with degenerate primers. Contamination events were excluded by usage of a negative control representing the same PCR mix but without DNA-template. The results of gel-electrophoresis demonstrated small accumulation of DOP-PCR-products generated from lampbrush chromosome loci (except for whole lampbrush chromosome W sample) (Additional file 1) that is mainly due to the extremely small amount of input material. At the same time, this amount of the primarily amplified products proved to be sufficient for subsequent steps of FISH-probes generation (Fig. 1).

Alternatively, we modified the dissection procedure to isolate and amplify the sequences from RNA-component of lampbrush chromosome marker structures (Fig. 1). As an example, we isolated RNA from the dissected material of the special loop on lampbrush chromosome 3. Specifically, lumpy loops from six bivalents were microdissected and then digested with DNAse I followed by reverse transcription step. Given that special types of RNAs have not been revealed in this marker structure, random hexamer primers were used for reverse transcription to synthesize cDNA from all RNAs in total RNA population. Then, as in case of DNA samples, reverse transcription products were amplified by PCR with degenerate primers (Fig. 1). As shown by gel-electrophoresis, the amount of the primarily amplified products was comparable with the one obtained after amplification of the whole lampbrush chromosome W.

To summarize, both DNA- and RNA-components of a single chromomere or lateral loops of lambrush chromosomes can be successfully isolated using needle-based microdossection and amplified or subjected to reverse transcription with further amplification.

### Generation of specific FISH-probes from the dissected lampbrush chromosome loci

**Fig. 3:**
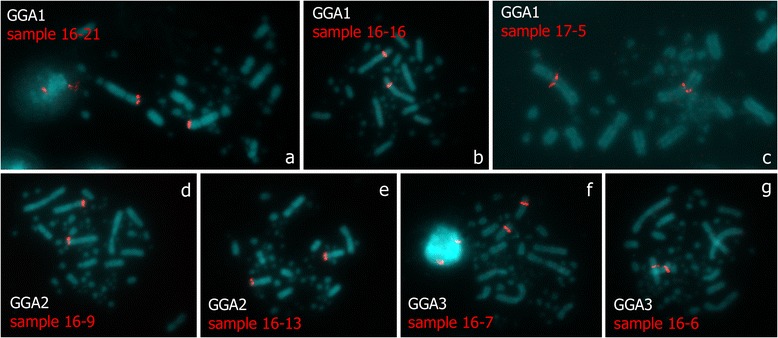
Verification of brightness and specificity of the DNA-probes by FISH on metaphase chromosomes. FISH with DNA-probes generated from different single LBC1 chromomeres (**a**-**c**), from «spaghetti marker» on LBC2 (**d**, **e**) and from «lumpy loops» on LBC3 (**f**, **g**). Names of samples are shown. Single pairs of hybridization signals (*red*) are detected on corresponding pairs of homologous chromosomes

Quality and specificity of FISH-probes generated from the dissected lampbrush chromosome regions was assessed by FISH on mitotic metaphase chromosomes (Fig. 1). Firstly, a painting probe prepared from the dissected material of lampbrush chromosome W was tested. FISH with the paint gave a bright and specific hybridization signal along the whole chromosome W in metaphase plates (Additional file 2). FISH with locus-specific probes gave two bright and distinct hybridization signals on corresponding pairs of homologous metaphase chromosomes in 100 % of analyzed samples (Fig. 3). In case of some probes, in particular those generated from the material of large DAPI-positive chromomeres, faint cross-hybridization in additional chromosomal regions could be detected under conditions of longer exposure time during image acquisition. Such cross-hybridization might be due to the bulk of retrotransposable elements spread along chicken chromosomes [46, 47].

**Fig. 4:**
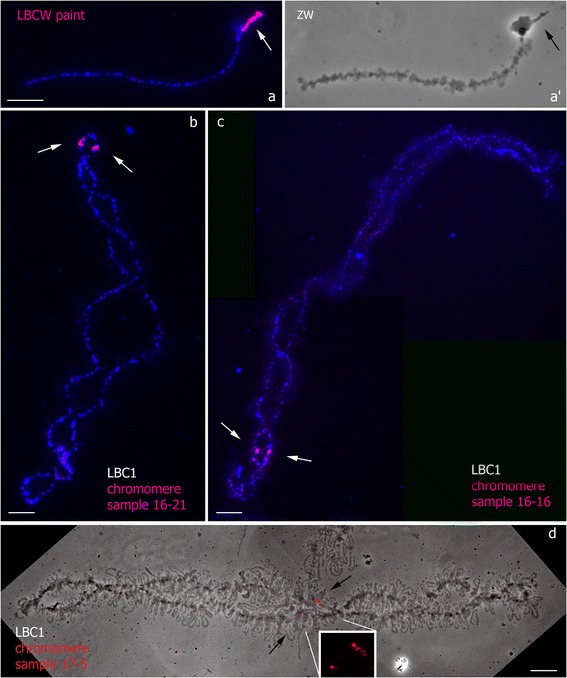
High-resolution FISH-mapping of DNA-probes generated from whole LBCW and individual chromomeres of LBC1 on lampbrush chromosomes. FISH with dissected material of whole LBCW (paint LBCW) on sex bivalent ZW (**a**), corresponding phase contrast image (*a’*). FISH with dissected material of individual chromomeres on LBC1 (**b**, **c**, **d**). Chromosomes are counterstained with DAPI (**a**, **b**, **c**); fluorescent signal is shown on the corresponding phase contrast image, insert shows enlarged chromosome region with fluorescent signals (**d**). Arrows point to chromosomal targets. Names of samples are shown. Scale bar = 10 μm

The dissected regions were mapped on chicken lampbrush chromosomes with high resolution. FISH on lampbrush chromosomes was carried out according to a DNA/DNA + RNA hybridization protocol so as to visualize both target DNA loci and RNA-transcripts of lateral loops. The LBCW painting probe hybridized to whole material of lampbrush chromosome W belonging to the sex bivalent ZW and representing an array of compact prominent loopless chromomeres (Fig. 4a, a’). The probes generated from various chromomeres, dissected from a single lampbrush chromosome 1, hybridized to corresponding loci on LBC1 (Fig. 4b, c, d). Remarkably, probes prepared from dissected material of marker prominent chromomeres gave bright massive hybridization signals in corresponding DAPI-positive loopless chromomeres (Fig. 4b, c). Alternatively, probes generated on the basis of small chromomeres were detected both in corresponding chromomeres and arising pairs of lateral loops (Fig. 4d).

**Fig. 5:**
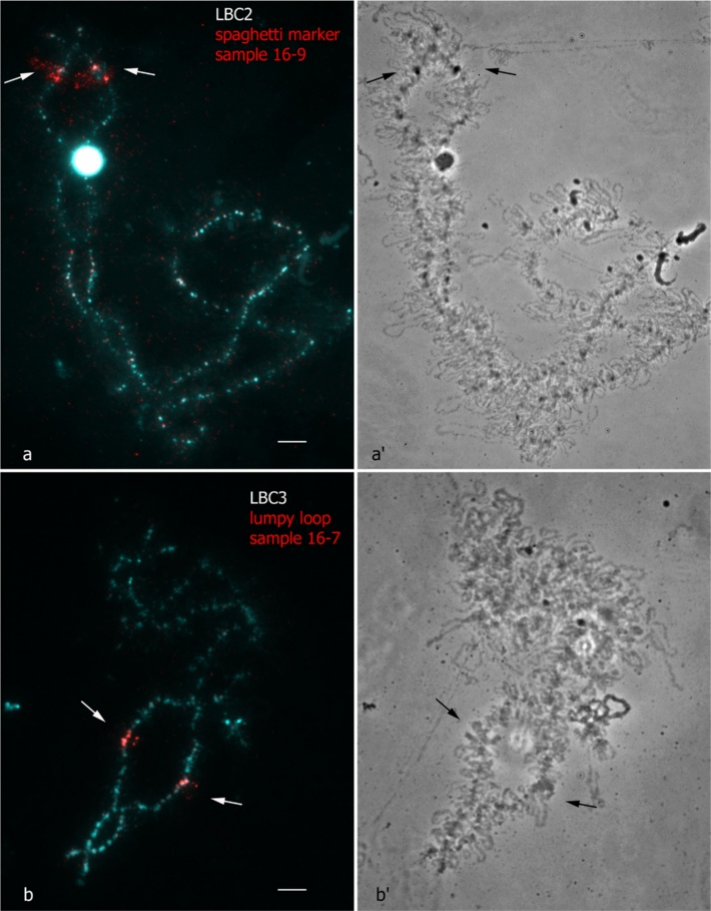
High-resolution FISH-mapping of DNA-probes generated from marker structures of LBC2 and LBC3 on lampbrush chromosomes. FISH with dissected material of spaghetti marker (SM) on LBC2 (**a**) and dissected material of lumpy loop (LL) on LBC3 (**b**). Chromosomes are counterstained with DAPI. Corresponding phase contrast images (*a’*, *b’*). Arrows point to SM and LL positions. Names of samples are shown. Scale bar = 10 μm

The DNA-probes gained from the dissected loci of marker structures – spaghetti marker (LBC2p) and lumpy loop (LBC3q) – effectively hybridized in immediate proximity to the landmark structures on corresponding lampbrush chromosomes (Fig. 5; Additional file 3). In particular, a fluorescent hybridization signal was detected in a pair of prominent chromomeres adjoining these structures clearly visible on corresponding phase-contrast images. In case of one sample, the probe hybridized to a transcriptional unit, turning to lumpy loop structure (Fig. 5b, b’).

**Fig. 6:**
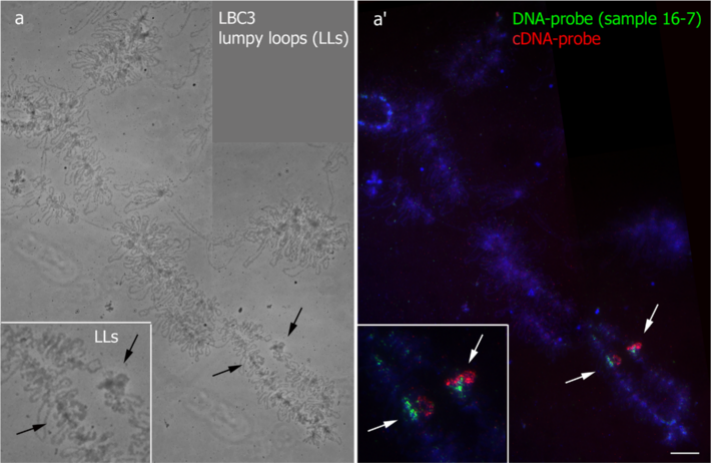
Co-hybridization of DNA- and cDNA-probes generated from the same chromosomal locus (LLs) on chicken lampbrush chromosome 3. Two-color FISH-mapping of DNA- (green signal) and cDNA-probes (red signal) to LL locus on LBC3 (*a’*); corresponding phase contrast image (**a**), inserts show enlarged chromosome region with LLs. Arrows point to LLs position. Names of samples are shown. Scale bar = 10 μm

In case of cDNA probes prepared on the basis of RNA-component of dissected lumpy loops, bright and specific hybridization signal was detected throughout the whole material (RNP-matrix) of special loops. Differentially labeled DNA- and RNA-based probes prepared from the same LL-locus co-hybridized and marked specifically the special loop (Fig. 6). At present, isolation of RNA from particular cell types by microdissection followed by real-time, microarray or RNA-seq analysis has become routine [48–51]. Here we offer for the first time an approach that provides a unique opportunity to obtain RNA samples from particular RNA-containing nuclear structures including RNP-matrix of individual transcription units.

In conclusion, lampbrush chromosome microdissection allows to prepare highly specific FISH-probes marking small target regions, thus representing a promising method for investigation of functionally significant elements of chromosomes.

### Mapping of the dissected chromosome regions to chicken genome assembly using high-throughput sequencing

Six microdissected DNA-samples were selected to be deciphered by high-throughput sequencing approach using Roche’s 454 platform. The following samples were sequenced: the material of two different chromomeres from lampbrush chromosome 1 – a large loopless chromomere (sample #16-16) and a small chromomere with arising loops (sample #17-5), two spaghetti marker samples from lampbrush chromosome 2 (#16-9 and #16-13) and two lumpy loop samples from lampbrush chromosome 3 (#16-6 and #16-7). To analyze the data obtained by high-throughput sequencing of DOP-PCR products, we optimized a workflow based on publicly available bioinformatic tools. For all samples, reliable sequence data was obtained and successfully aligned to chicken reference genome assembly. Thus, the output proved to be similar to a yield of mitotic chromosome microdissection technique and higher as compared to a success rate in a recently developed approach of so-called “nano-dissection”. That is, dissection of DNA from sub-nuclear domains using a scanning electron microscope yielded *bona fide* DNA sequences only in 16 % of cases [52].

For the most successfully sequenced samples, a total number of reads was 12 356 (large chromomere, sample #16-16) and 4 796 (lumpy loops, sample #16-7) that is comparable with a harvest of high-throughput sequencing of microdissected metaphase chromosomes where 6–10 chromosomal arm copies were used as starting material [23, 24]. The maximization of read’s number was not our priority, and only the reads of a good quality were taken into analysis. About 63 – 82 % of obtained sequencing reads was mapped to chicken reference genome assembly (ICGSC Gallus_gallus-4.0/galGal4) with the vast majority of reads having their hits on target chromosomal regions. High content of interspersed repetitive DNA elements distributed throughout chicken chromosomes [46, 47] as well as any annotation imperfections might be largely responsible for the presence of off-target hits and a portion of unmapped reads.

**Fig. 7:**
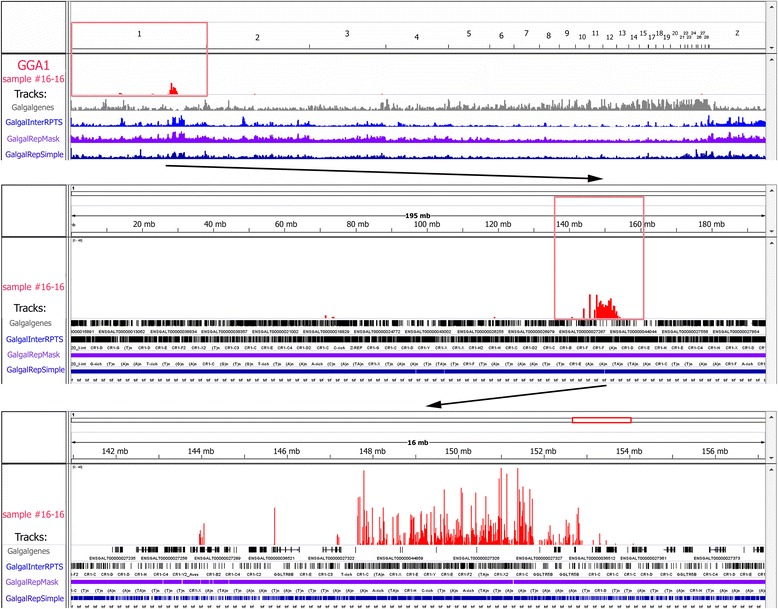
Mapping of the dissected lampbrush chromosome 1 region to chicken genome using high-throughput sequencing. Visualization of sequencing data via the «Integrative Genomics Viewer» (IGV, [43, 44]) on example of a single LBC1 chromomere (sample #16-16). Sequencing reads successfully aligned to chicken genome are depicted in red and outlined by frames. An upper panel **-** «all chromosomes» view; a middle panel – «the chromosome» view; a lower panel **–** a zoomed target region. Imported UCSC-tracks containing information on chicken genes (gray track) and different types of repetitive sequences (blue, purple, dark blue tracks) are shown. Based on the results of mapping, precise genomic position, extent and sequence content were determined for the dissected chromomeres

**Fig. 8:**
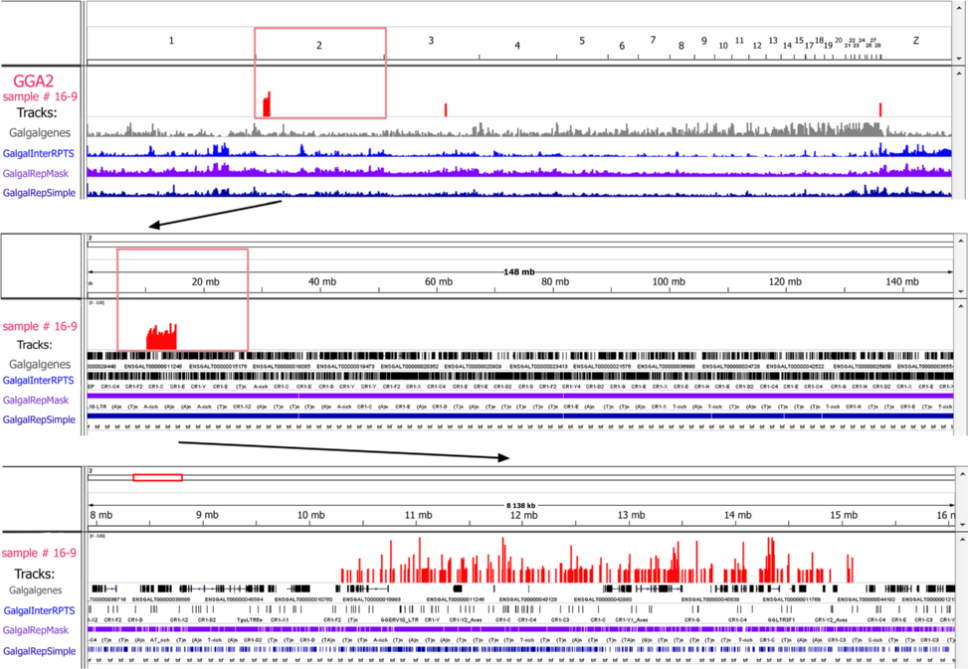
Mapping of the dissected lampbrush chromosome 2 region to chicken genome using high-throughput sequencing. Visualization of sequencing data via the «Integrative Genomics Viewer» (IGV) on example of «Spaghetti marker» locus on LBC2 (sample #16-9). All indications are the same as in Fig. 7. Based on the results of mapping, precise genomic position, extent and sequence content were determined for the dissected SM locus

**Fig. 9:**
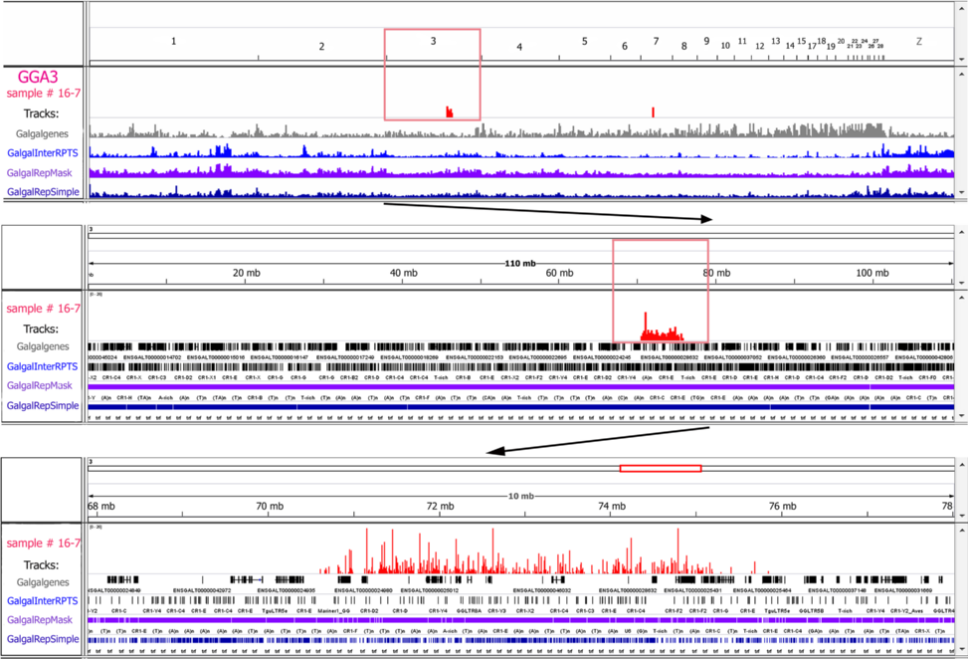
Mapping of the dissected lampbrush chromosome 3 region to chicken genome using high-throughput sequencing. Visualization of sequencing data via the «Integrative Genomics Viewer» (IGV) on example of «Lumpy loop» locus on LBC3 (sample #16-7). All indications are the same as in Fig. 7. Based on the results of mapping, precise genomic position, extent and sequence content were determined for the dissected LL locus

As a result of alignment experiments, the dissected chromosomal regions were assigned to chicken genomic coordinates with high resolution (Figs. 7, 8 and 9). The overwhelming majority of on-target reads obtained from the dissected large chromomere (Fig. 4c) was mapped to a distal part of chromosome 1 (GGA1) q-arm, specifically, to 148-153-Mb region of GGA1 sequence assembly (Fig. 7). Hence, according to this assessment, the amount of DNA in such massive chromomeres can be evaluated as about 4–5 Mb. DNA in the investigated large chromomere proved to be gene-poor as compared to bordering regions (Fig. 7). Moreover, the chromomere contained higher amount of repetitive sequences both simple repeats and interspersed retrotransposable elements (including chicken widespread LINE СR1) (Fig. 7). The results suggest that there is an increased ratio of interspersed repeats to unique sequences in DNA of large chromomeres of lampbrush chromosome arms.

In case of a small microdissected chromomere (Fig. 4d), on-target reads spanned a genomic region of only 2 Mb in size at 79.5 – 81.5 Mb position on GGA1 sequence assembly. This region corresponds to a proximal part of LBC1 q-arm that is localized several chromomeres below the centromere and has been described in detail using chicken BAC-clones in our previous study [53]. The result is in a good agreement with previous evaluations of average DNA-content in a single chromomere of chicken lampbrush macrochromosomes [45]. In contrast to the large marker chromomere, there was no significant difference in gene density between sequences from the small chromomere and adjacent regions; it did not show visible enrichment with repetitive sequences as well. Together with FISH results, NGS data gives evidence of the discrepancy in DNA content and transcriptional status between large and small chromomeres. For comparison, in recent studies, a combination of multiple cytogenetic, molecular-genetic and bioinformatic methods was applied to explore the genomic characteristics of particular chromosomal regions (bands and interbands) of giant polytene chromosomes of *Drosophila melanogaster* [54, 55]. Here, we demonstrate that lampbrush chromosome microdissection represents an alternative appropriate tool for exhaustive investigation of genomic organization of cytologically distinct chromosomal regions.

We also analyzed and compared the results of high-throughput sequencing for two spaghetti marker samples dissected from two different LBC2 bivalents. The first dissected locus was aligned to chromosome 2 (GGA2) p-terminus and extended approximately from 10.3 Mb to 15 Mb on GGA2 sequence assembly thus being about 4.5-5 Mb in length (Fig. 8). The size of the dissected region evaluated upon the sequencing results is consistent with FISH data, where hybridization signal from the probe was detected in two chromomeres flanking spaghetti marker (Fig. 5a, a’). With regard to sequence content, the region could be visually subdivided into two subregions (Fig. 8). That is, a subregion at 10.3-12.7 Mb position contains just a few gene annotations and is enriched with repetitive sequences while another one expanded from 12.7 Mb to 15 Mb is rich in genes and does not contain so many repetitive tracks. The second sequenced locus proved to be more extended and mapped to 9 – 18.5 Mb position on GGA2 sequence assembly (Additional file 4). It is supported by FISH results according to which hybridization signals were detected in two prominent chromomeres on both sides from spaghetti marker structure (Additional file 3 a, a’). Taken into account FISH and high-throughput sequencing results, the locus of spaghetti marker formation can be narrowed down and assigned to the region at 11–13 Mb position on GGA2. It is worth noting that there are several joints within the contig NW_003763661.1 at this position in the current version of GGA2 sequence assembly [56]. The joints are represented by non-sequenced tracks usually corresponding to some repetitive sequences that could underlie the «spaghetti marker» chromosome-associated nuclear structure.

Two DNA samples dissected from lumpy loops locus on lampbrush chromosome 3 were also deciphered by high-throughput sequencing and aligned against chicken genome sequence assembly. The first dissected chromosomal region was mapped to 70.5-75.5 Mb position with the majority of reads being aligned to 71–75 Mb region (Fig. 9), and the second one – to 69.5 – 74.5 Mb position (Additional file 5). Within this locus there is a subregion (72–74.5 Mb position) that comprises only single gene annotations but is enriched with repeats in contrast to flanking sequences (Fig. 9; Additional file 5). As in case of spaghetti marker locus on chromosome 2, the contig NW_003763720.1 in GGA3 sequence assembly [56] contains undeciphered tracks at 72.4 and 73.6 positions. Such a complicated region could be a locus of lumpy loops seeding. The results shed light on the precise genomic context of the distinctive cytological structures from which the probes were derived.

## Conclusions

Here we show that lampbrush chromosome microdissection allows to generate locus-specific FISH-probes and to assign dissected regions to reference genome by high-throughput sequencing (Fig. 1). The results obtained demonstrate that generated FISH-probes are highly specific and can mark the chromosomal loci as small as individual chromomeres and lateral loops. The protocol also provides an opportunity to define precisely the genomic position, extent and sequence content of dissected regions, and thus makes it possible to correlate a particular loop (or a group of loops) with known genes. Moreover, given that certain lateral loops represent single transcription units, lampbrush chromosome microdissection could clarify what sequences are being transcribed by single loops and give us a deeper insight into mechanisms of transcription. To conclude, the developed microdissection approach offers great prospects for detailed exploration of functionally significant chromosomal regions including individual transcription units.

## Additional files

Additional file 1:
**Primary amplification of microdissected samples by DOP-PCR.** Description of data: The result of DNA gel electrophoresis of dissected samples primarily amplified by DOP-PCR (examples). Lines: 1, 2 – single chromomeres dissected from LBC1; 3, 4 – SMs samples dissected from LBC2; 5, 6 – LLs samples dissected from LBC3; 7 – whole LBCW; 8 – negative control (collection drop without dissected material); 9 – 100 bp ladder. Samples were run in 1 % agarose gel (TIF 5958 kb) Additional file 2:
**Verification of brightness and specificity of the whole-chromosome W paint by FISH on metaphase chromosomes.** Description of data: FISH with the DNA-probe generated from dissected material of lampbrush chromosome W. Bright and specific hybridization signal (red) is detected along the whole chromosome W. (TIF 6575 kb) Additional file 3:
**High-resolution FISH-mapping of DNA-probes generated from marker structures of LBC2 and LBC3 on lampbrush chromosomes (additional samples).** Description of data: FISH with dissected material of spaghetti marker (SM) on LBC2 (a) and dissected material of lumpy loop (LL) on LBC3 (b). Chromosomes are counterstained with DAPI. Corresponding phase contrast images (a’, b’). Arrows point to SM and LL positions. Names of samples are shown. Scale bar = 10 μm. (TIF 19826 kb) Additional file 4:
**Mapping of the dissected lampbrush chromosome 2 regions to chicken genome using high-throughput sequencing (additional sample).** Description of data: Visualization of sequencing data via the «Integrative Genomics Viewer» (IGV) on the example of «Spaghetti marker» locus on LBC2 (sample #16-13). Sequencing reads successfully aligned to chicken genome are depicted in red and outlined by frames. An upper panel - «all chromosomes» view; a middle panel – «the chromosome» view; a lower panel – a zoomed target region. Imported UCSC-tracks containing information on chicken genes (gray track) and different types of repetitive sequences (blue, purple, dark blue tracks) are shown. Based on the results of mapping, precise genomic position, extent and sequence content were determined for the dissected SM locus. (TIF 33589 kb) Additional file 5:
**Mapping of the dissected lampbrush chromosome 3 regions to chicken genome using high-throughput sequencing (additional sample).** Description of data: Visualization of sequencing data via the «Integrative Genomics Viewer» (IGV) on the example of «Lumpy loop» locus on LBC3 (sample #16-6). All indications are the same as in Additional file 4. Based on the results of mapping, precise genomic position, extent and sequence content were determined for the dissected LL locus. (TIF 32474 kb)

## Abbreviations

cDNA: Complementary DNA
DOP-PCR: Degenerate oligonucleotide-primed PCR
FISH: Fluorescent in situ hybridization
GGA: Chicken (*Gallus gallus*) chromosome
IGV: Integrative genomics viewer
LBC: Lampbrush chromosome
LL: Lumpy loop
NGS: Next-generation sequencing
RT: Reverse transcription
SM: Spaghetti marker
SSC: Saline-sodium citrate buffer
