# Supplementary figures and images for: Microdissection of lampbrush chromosomes as an approach for generation of locus-specific FISH-probes and samples for high-throughput sequencing
Source: BMC Genomics. 2016 Feb 20;17:126. doi: 10.1186/s12864-016-2437-4 (PMC4761191; doi:10.1186/s12864-016-2437-4)

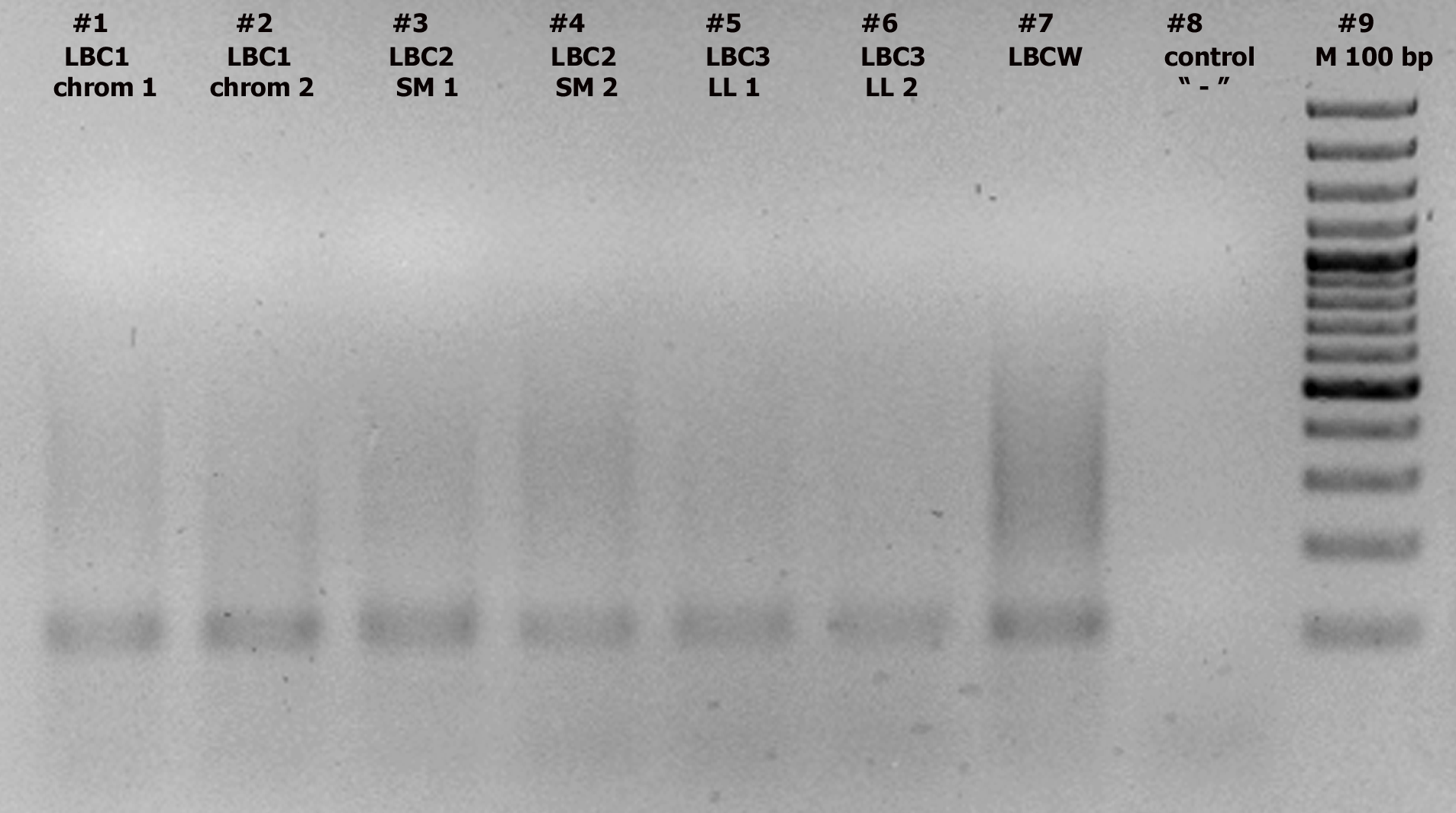

Supplement: Additional file 1: — Primary amplification of microdissected samples by DOP-PCR. Description of data: The result of DNA gel electrophoresis of dissected samples primarily amplified by DOP-PCR (examples). Lines: 1, 2 – single chromomeres dissected from LBC1; 3, 4 – SMs samples dissected from LBC2; 5, 6 – LLs samples dissected from LBC3; 7 – whole LBCW; 8 – negative control (collection drop without dissected material); 9 – 100 bp ladder. Samples were run in 1 % agarose gel (TIF 5958 kb) [file 12864_2016_2437_MOESM1_ESM.tif]

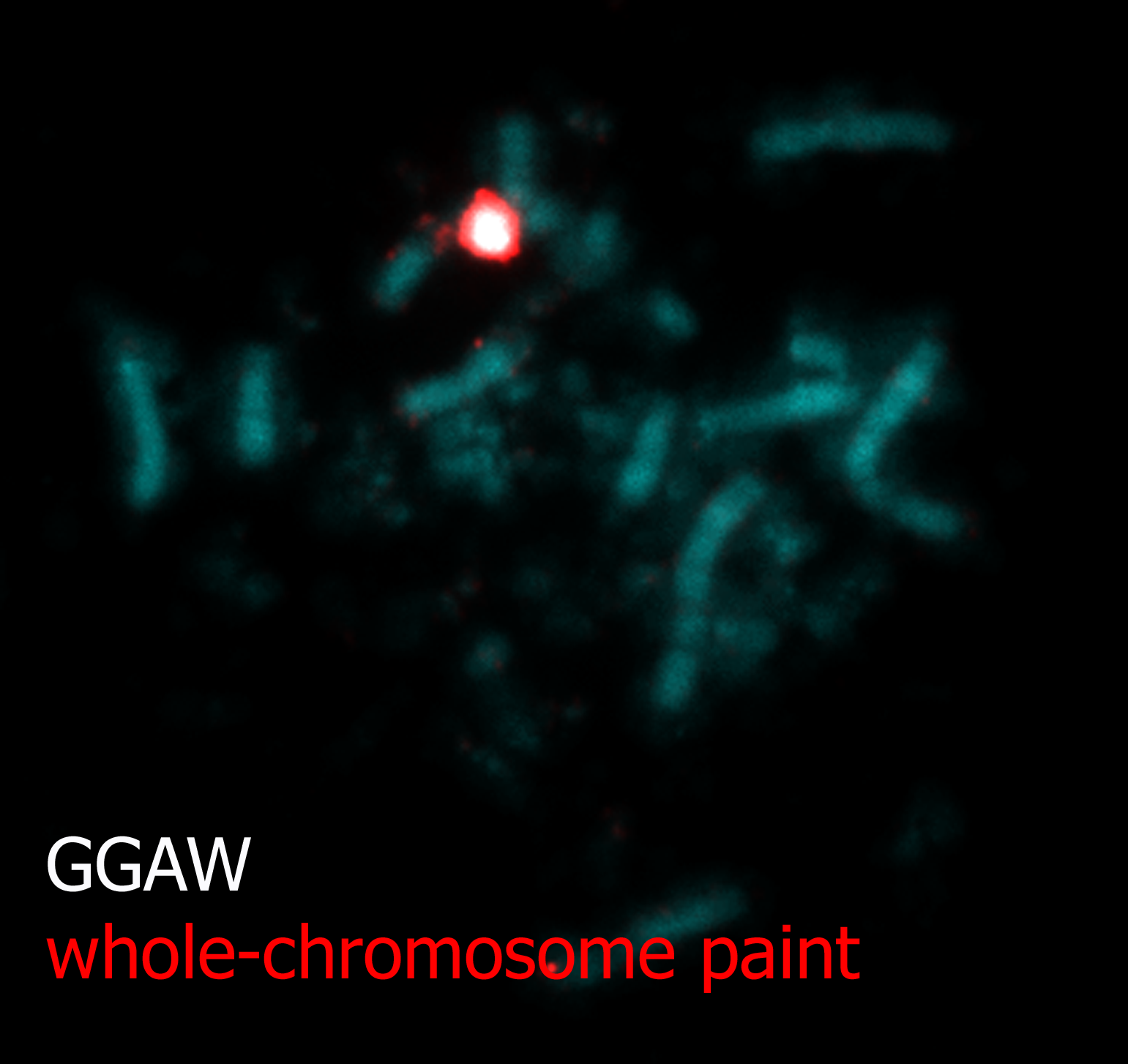

Supplement: Additional file 2: — Verification of brightness and specificity of the whole-chromosome W paint by FISH on metaphase chromosomes. Description of data: FISH with the DNA-probe generated from dissected material of lampbrush chromosome W. Bright and specific hybridization signal (red) is detected along the whole chromosome W. (TIF 6575 kb) [file 12864_2016_2437_MOESM2_ESM.tif]

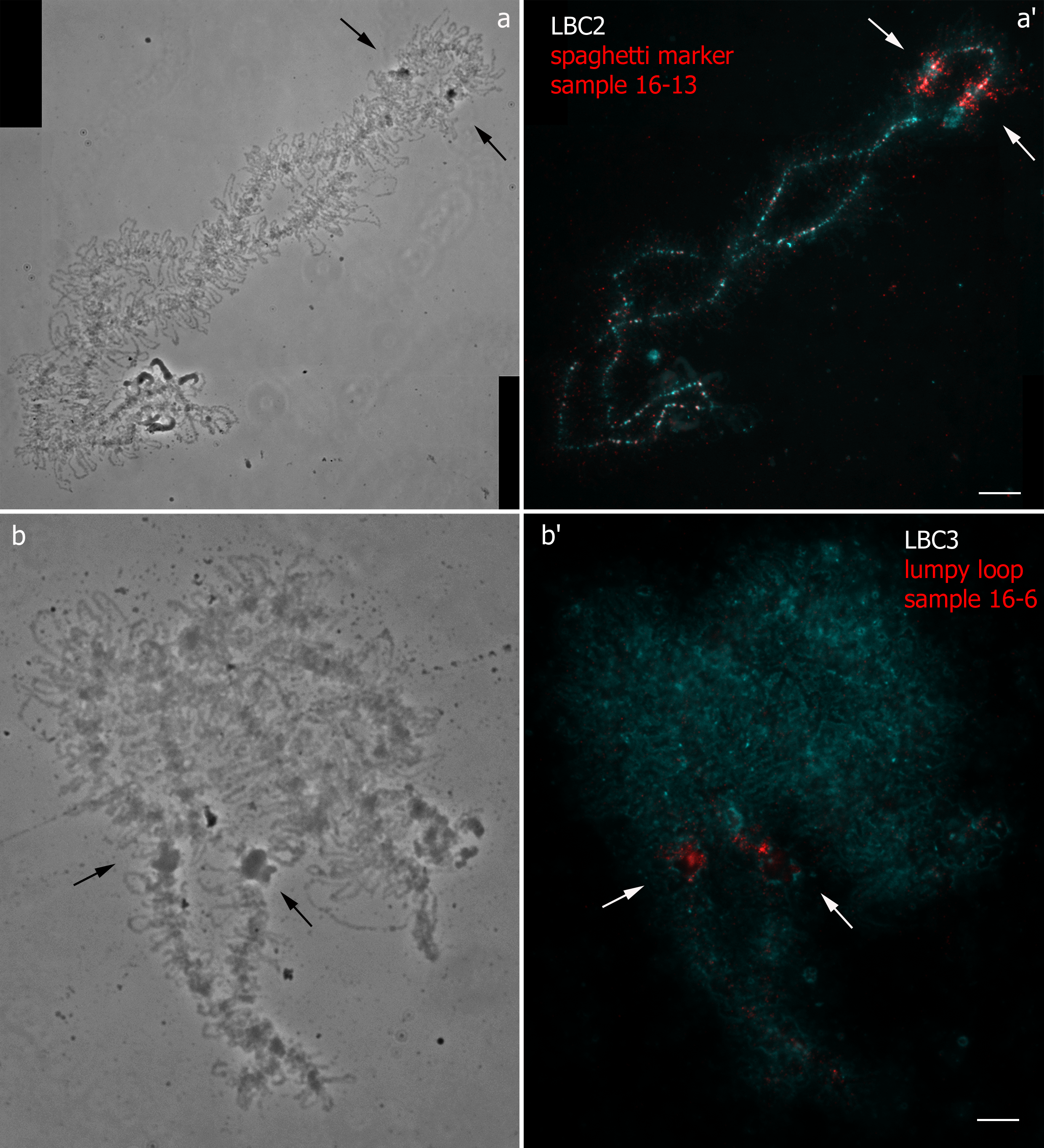

Supplement: Additional file 3: — High-resolution FISH-mapping of DNA-probes generated from marker structures of LBC2 and LBC3 on lampbrush chromosomes (additional samples). Description of data: FISH with dissected material of spaghetti marker (SM) on LBC2 (a) and dissected material of lumpy loop (LL) on LBC3 (b). Chromosomes are counterstained with DAPI. Corresponding phase contrast images (a’, b’). Arrows point to SM and LL positions. Names of samples are shown. Scale bar = 10 μm. (TIF 19826 kb) [file 12864_2016_2437_MOESM3_ESM.tif]

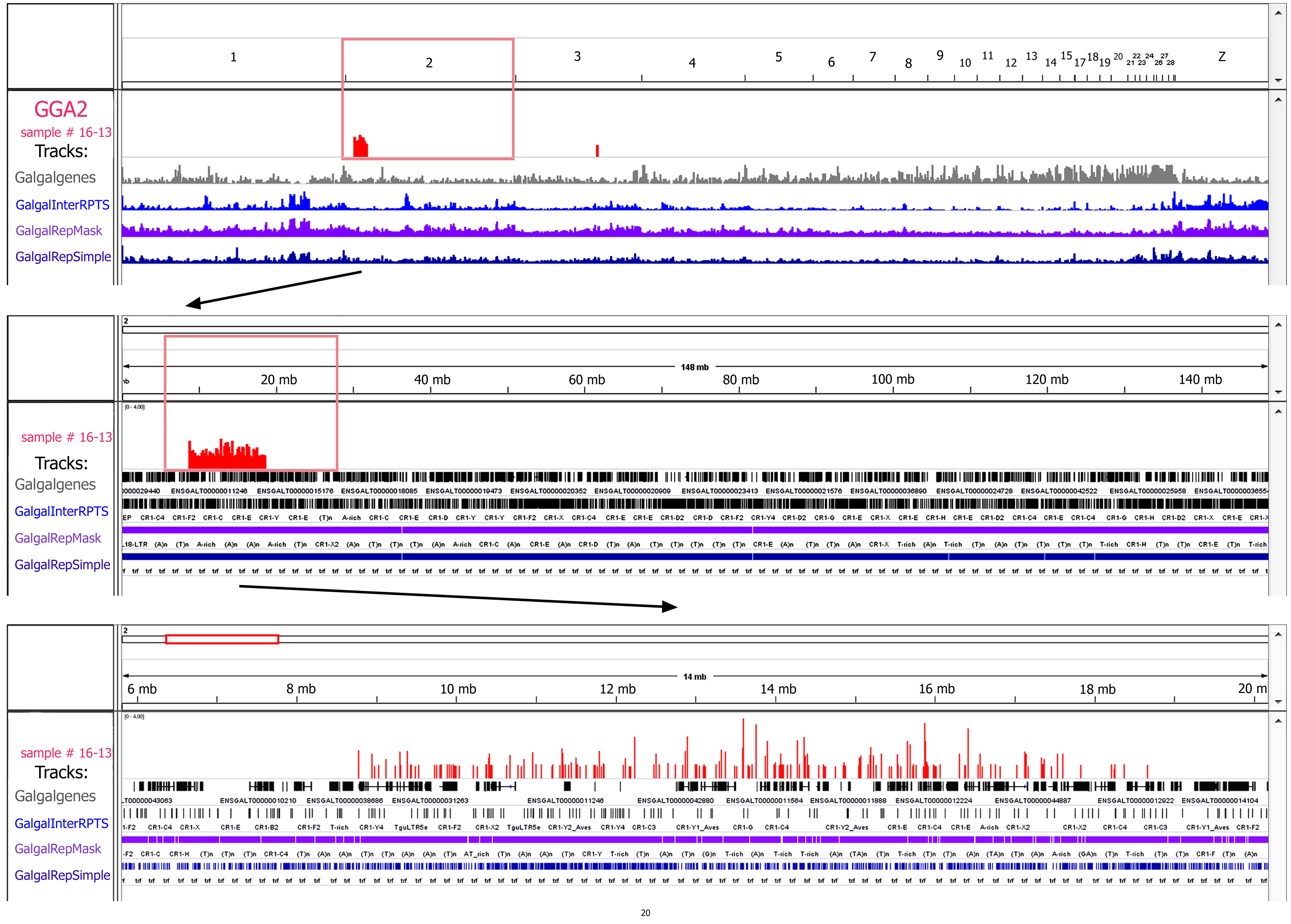

Supplement: Additional file 4: — Mapping of the dissected lampbrush chromosome 2 regions to chicken genome using high-throughput sequencing (additional sample). Description of data: Visualization of sequencing data via the «Integrative Genomics Viewer» (IGV) on the example of «Spaghetti marker» locus on LBC2 (sample #16-13). Sequencing reads successfully aligned to chicken genome are depicted in red and outlined by frames. An upper panel - «all chromosomes» view; a middle panel – «the chromosome» view; a lower panel – a zoomed target region. Imported UCSC-tracks containing information on chicken genes (gray track) and different types of repetitive sequences (blue, purple, dark blue tracks) are shown. Based on the results of mapping, precise genomic position, extent and sequence content were determined for the dissected SM locus. (TIF 33589 kb) [file 12864_2016_2437_MOESM4_ESM.tif]

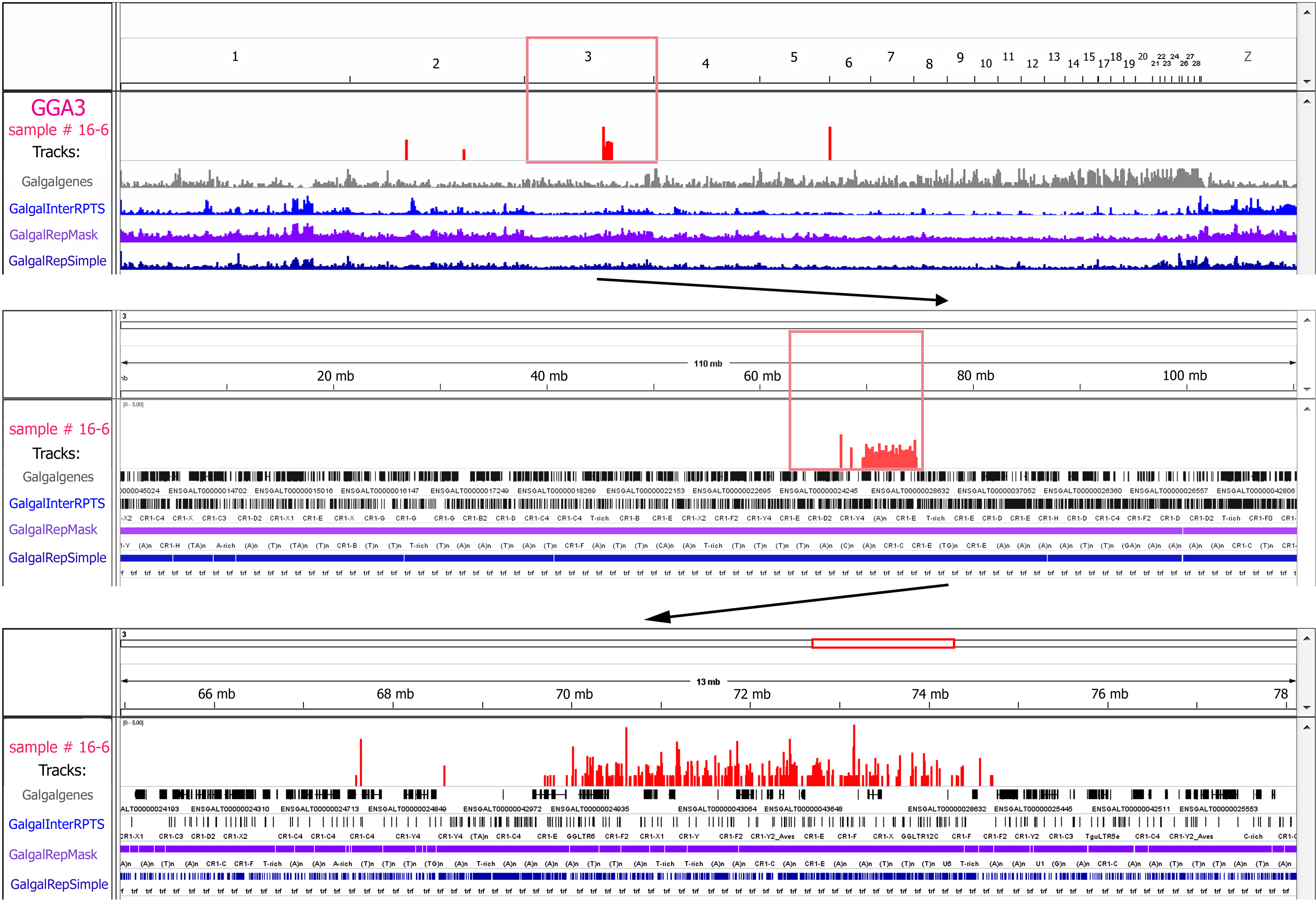

Supplement: Additional file 5: — Mapping of the dissected lampbrush chromosome 3 regions to chicken genome using high-throughput sequencing (additional sample). Description of data: Visualization of sequencing data via the «Integrative Genomics Viewer» (IGV) on the example of «Lumpy loop» locus on LBC3 (sample #16-6). All indications are the same as in Additional file 4. Based on the results of mapping, precise genomic position, extent and sequence content were determined for the dissected LL locus. (TIF 32474 kb) [file 12864_2016_2437_MOESM5_ESM.tif]
